# Supplementary material for: Sustained CREB phosphorylation by lipid-peptide liquid crystalline nanoassemblies
Source: Commun Chem. 2023 Nov 6;6:241. doi: 10.1038/s42004-023-01043-9 (PMC10628290; doi:10.1038/s42004-023-01043-9)
Supplement: Supplementary file 2 — Supplementary Information [file 42004_2023_1043_MOESM2_ESM.pdf]

Supplementary Information

Sustained CREB Phosphorylation by Lipid-Peptide Liquid Crystalline Nanoassemblies

Yu Wu[1], Borislav Angelov[2,], Yuru Deng[3], Takehiko Fujino[4,], Md Shamim Hossain[4], Markus Drechsler[5], and Angelina Angelova[1,*]*

[1]Université Paris-Saclay, CNRS, Institut Galien Paris-Saclay, F-91400 Orsay, France,

[2]Extreme Light Infrastructure ERIC, CZ-25241 Dolni Brezany, Czech Republic,

[3]Wenzhou Institute, University of Chinese Academy of Sciences, No.1, Jinlian Road, Longwan District, Wenzhou, Zhejiang 325001, China,

[4]Institute of Rheological Functions of Food, 2241-1 Kubara, Hisayama-cho, Kasuya-gun, Fukuoka 811-2501, Japan,

[5]Keylab "Electron and Optical Microscopy", Bavarian Polymerinstitute (BPI), University of Bayreuth, Universitätsstrasse 30, D-95440 Bayreuth, Germany.

Table S1. Compositions of natural scallop-derived scPL70- and PUFA-plasmalogen-based LNP nanoformulations of vesicular (PL-V), cubosome (PL-C), and hexosome (PL-H) types. The color codes correspond to the SAXS patterns presented in Figure 3. The abbreviations denote the ingredients PL-DHA-PE: 1-(1Z-octadecenyl)-2-docosahexaenoyl-sn-glycero-3-phosphoethanolamine, scPL70: scallop-derived plasmalogen extract, MO: monoolein, and TPGS-PEG_{1000}: D-α-tocopherol polyethylene glycol-1000 succinate.

Sample name	Composition
**PL-V**	scPL70 (0.15g)/Vitamin E (0.02g)/TPGS-PEG_{1000}(0.04g)
	scPL70 (0.15g)/Q_{10} (0.004g)/TPGS-PEG_{1000}(0.04g)
**PL-C**	MO(0.1g)/scPL70 (0.04g)/Vitamin E (0.02g)/TPGS-PEG1000(0.04g)
	MO(0.03g)/PL-DHA-PE (0.012g)/Vitamin E (0.004g)/Q_{10}(0.0008g)/TPGS-PEG_{1000}(0.008g)
**PL-H**	MO(0.03g)/PL-DHA-PE (0.012g)/Vitamin E (0.004g)/TPGS-PEG_{1000}(0.008g)
	MO(0.03g)/scPL70 (0.15g)/Vitamin E (0.02g)/TPGS-PEG_{1000}(0.04g)

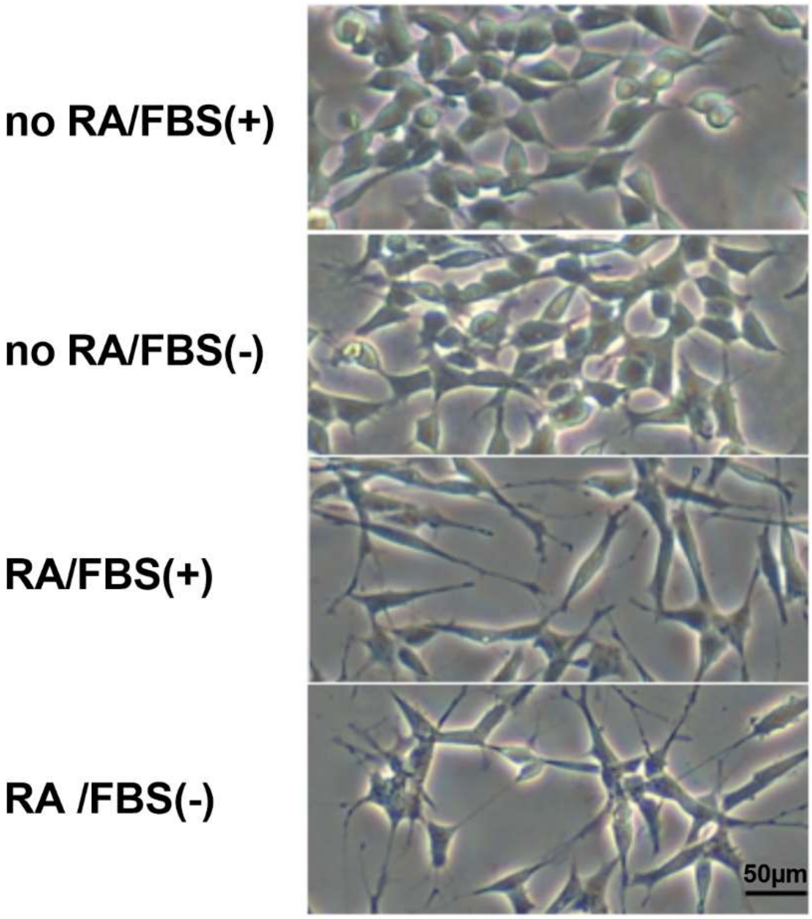

Figure S1. Optical micrographs of SH-SY5Y cellular morphologies showing the effect of starvation before and after treatment by retinoic acid (RA 10µM) for differentiation into a neuronal phenotype. The cell culture conditions correspond to

- non-differentiated cells (no RA) grown in serum-containing medium (FBS+);
- non-differentiated cells (no RA) exposed for 24h to FBS-free medium (FBS-), *i.e.* 24h starvation of non-differentiated cells;
- RA-differentiated cells for 5 days in FBS-complete medium (RA/(FBS(+)); and
- RA-differentiated cells (5 days) that are further exposed for 24h to RA-containing FBS-free medium (RA/FBS (-)), *i.e.* 24h starvation in RA-containing medium.

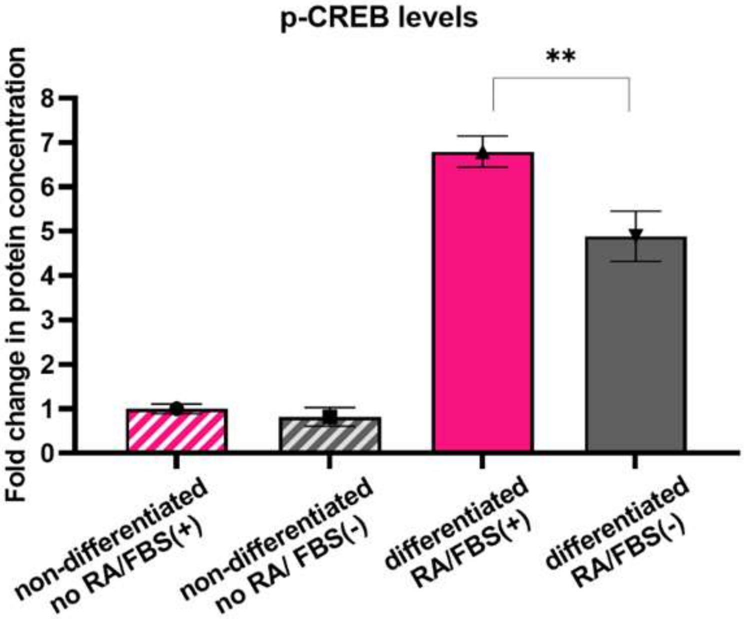

Figure S2. Effect of SH-SY5Y cellular differentiation and starvation on the fold changes in phosphorylated CREB (pCREB) levels before the oxidative damage by 6-OHDA. The cell culture conditions correspond to:

- non-differentiated SH-SY5Y cells grown in a medium containing FBS, *i.e.* no RA/FBS(+);
- non-differentiated SH-SY5Y cells exposed for 24h to FBS-free medium, i.e. no RA/FBS(-);
- RA-differentiated SH-SY5Y cells grown in FBS-containing complete medium for 5 days, *i.e.* RA/FBS(+) ;
- RA-differentiated SH-SY5Y cells exposed for 24h to FBS-free medium, *i.e.* RA/FBS(-).

Phosphorylated CREB was quantified by ELISA (n = 3). Using the Prism software, the Dunnett test was used for multiple group comparison, while the Student t-test was employed for comparing two groups. Statistical significance is indicated as * $P \leq 0.05$, ** $P \leq 0.01$, and *** $P \leq 0.001$.

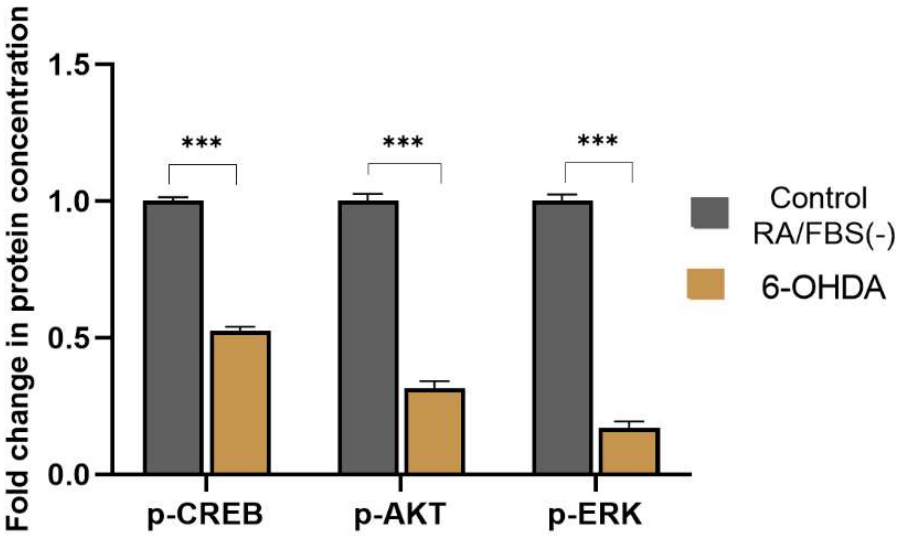

Figure S3. Fold changes in the phosphorylation levels of CREB, AKT, and ERK proteins following 6-OHDA (30 min) treatment of 24h-starved differentiated SH-SY5Y cells, quantified by ELISA assays. The control RA/FBS(-) corresponds to RA-differentiated SH-SY5Y cells exposed for 24h to an FBS-free medium (n=3). Using the Prism software, the Dunnett test was used for multiple group comparison, while the Student t-test was employed for comparing two groups. Statistical significance is indicated as * $P \leqslant 0.05$, ** $P \leqslant 0.01$, and *** $P \leqslant 0.001$.

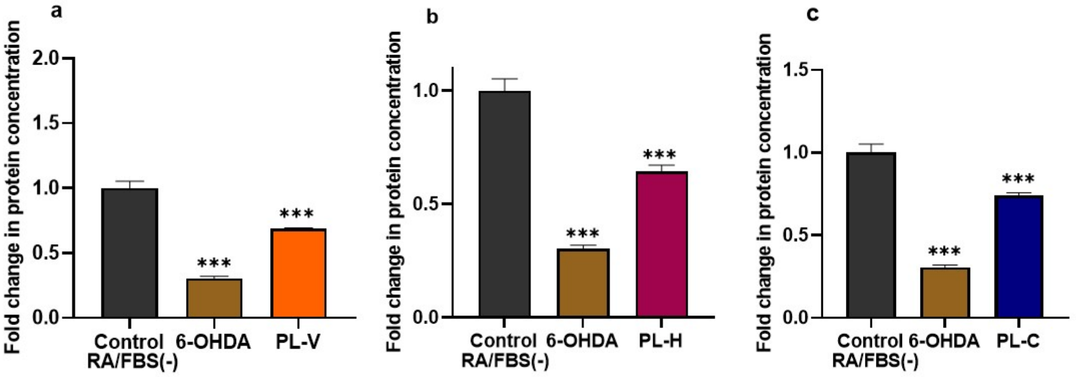

Figure S4. Fold changes in the phosphorylation levels of CREB protein (pCREB) after exposure to 6-OHDA (30 min, 200 μM) or LNPs (24h, 10 μM) incubation. Nanoformulations of PL-V, PL-H, and PL-C are compared. Protein phosphorylation levels were quantified using an ELSA assay with regard to an RA/FBS(-) control sample (*i.e.* differentiated SH-SY5Y cells deprived of serum for 24h) (n=3). Using the Prism software, the Dunnett test was used for multiple group comparison, while the Student t-test was employed for comparing two groups. Statistical significance is indicated as * P⩽0.05, ** P⩽0.01, and *** P⩽0.001.

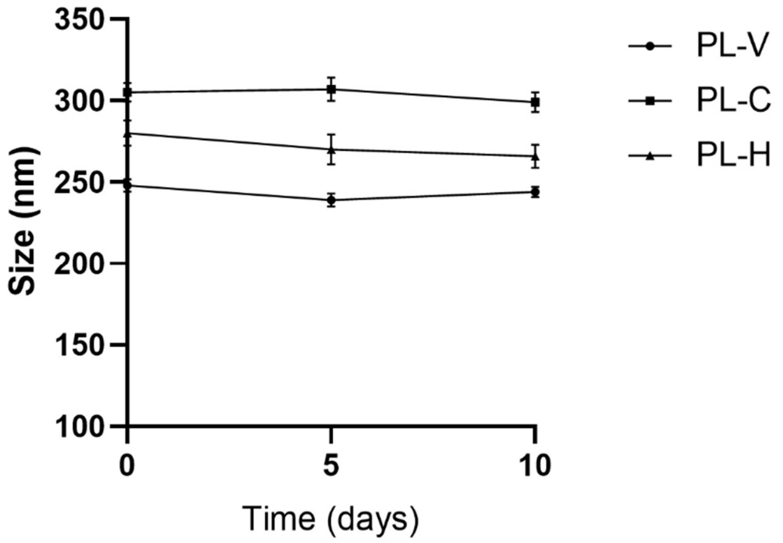

Figure S5. Nanoparticle sizes of the investigated three plasmalogen-based formulations determined by quasi-elastic light scattering as a function of time at 25°C (n = 3).
