# Supplementary material for: Sustained CREB phosphorylation by lipid-peptide liquid crystalline nanoassemblies
Source: Commun Chem. 2023 Nov 6;6:241. doi: 10.1038/s42004-023-01043-9 (PMC10628290; doi:10.1038/s42004-023-01043-9)
Supplement: Supplementary file 3 — Description of Additional Supplementary File [file 42004_2023_1043_MOESM3_ESM.pdf]

Description of Additional Supplementary Files

File name: Supplementary Data 1

Description: Data sets underlying Figures 5, 6, 7, and 8.
